# ID 297 - Contexto da Judicialização no Distrito Federal de Tecnologias em Saúde Não Recomendadas pela Conitec

**DOI:** 10.5327/2237-9622.2025.v34s1.297

**Published:** 2025-11-25

**Authors:** Viviane Corrêa de Almeida Fernandes, Samara Haddad Simões Machado, Débora Santos Lula Barros

**Keywords:** tecnologia em saúde, judicialização da saúde, custos de medicamentos

## Abstract

**Introdução:**

O Sistema Único de Saúde (SUS) garantiu o acesso integral e universal ao cuidado à saúde. O acesso ao medicamento pelo SUS ainda apresenta percalços. Ademais, o acesso ao medicamento é garantido em parte pela atuação da Comissão Nacional de Incorporação de Tecnologias em Saúde no SUS (Conitec), que avalia critérios como segurança, eficácia, efetividade, eficiência e impacto orçamentário, ético, social e ambiental. Todavia, o não acesso ao medicamento fortalece a judicialização da saúde. O objetivo deste trabalho é discutir o contexto judicial do sistema de gestão estadual do Distrito Federal (DF) de tecnologias não recomendas à incorporação pela Conitec.

**Método:**

Trata-se de um estudo primário, retrospectivo pela análise econômica de tecnologias em saúde (TS) judicializadas no DF. Os dados utilizados foram concedidos via Lei de Acesso à Informação (Lei n.º 12.527, de 18 de novembro de 2011) e sistema de gestão SIS-Materiais e tratados pelo programa Microsoft Excel.

**Resultados:**

Hoje os principais objetos de judicialização no DF são ocrelizumabe, pirfenidona, ustequinumabe, dupilumabe, riociguate e nintedanibe. Todas esses são medicamentos não padronizados e com recente decisão inicial de não recomendação de incorporação no SUS. A análise pela Conitec de cada TS foi publicada em Relatório a Sociedade, em 2018, para a pirfenidona e nintedanibe; em 2020, ocrelizumabe e ruxolitinibe; em 2022, para riociguate; e, em 2024, para dupilumabe, sendo esses responsáveis por consumir em torno de 26,5% do orçamento gasto com aquisição de medicamentos judiciais nos últimos quatro anos. Em 2020, houve gasto de R$ 34.049.578,27 anuais no DF com judicialização, chegando a R$ 60.560.455,75 em 2023, um aumento de 75%.

**Conclusão:**

O aumento progressivo dos gastos com aquisição por demandas judiciais torna a gestão da saúde mais desafiadora, já que a aquisição ocorre por dispensa de licitação. A dispensa de licitação é algo que deve ocorrer de maneira excepcional, visto que os preços praticados são maiores do que pelo processo regular. Uma série de eventos também pode ter influenciado o aumento do gasto progressivo com TS, como, por exemplo: a crise econômica mundial em 2022; a pandemia da covid-19; a Guerra Russo-Ucraniana; crises de energia e de alimentos; aumento de inflação; assim como emergências climáticas. Nesse cenário, houve desaceleramento do crescimento econômico mundial, conforme relatório das Nações Unidas . Todos esses fatores influenciam o ajuste de preços de insumos farmacêuticos no mercado e no valor da TS. O aumento dos preços das TS pode justificar o aumento progressivo dos gastos anuais com judicialização. O não fornecimento imediato da decisão judicial resulta no sequestro de verba por determinação judicial, causando danos indiretos ao erário público, por alterar o planejamento de recursos da saúde. A não recomendação de incorporação da TS desencadeia uma séria de processos judiciais ao Estado na prerrogativa de que o acesso é direito de todos e dever do Estado. Considerando que os medicamentos de maior importância econômica no DF estão relacionados a medicamentos de alto custo, haveria grande possibilidade dessas TS, ao ser incorporadas ao SUS, pertencerem ao Componente Especializado da Assistência Farmacêutica (Ceaf), tendo a União responsabilidade financeira maior frente aos entes estaduais. Atém-se em dizer que a não incorporação avalia a custo-efetividade, sendo as análises aqui apresentadas limitadas à análise orçamentária. É notório que há um impacto importante na gestão de recursos estaduais com a não incorporação da TS no SUS.

